# Peace in Georgia, at Last

**Published:** 1872-04

**Authors:** 


### PEACE IN GEORGIA, AT LAST.

It will be seen from the following action had at the late meeting of the Georgia Medical Association, at Columbus, Ga., tnat the professional war in this State has been ended and peace has (at last) been declared. Dr. Logan introduced the following:

*Resolved*, That the following construction shall be placed upon the action of the Association at Americus, namely:

All intention to charge upon the members of the Association personal motives in the then pending controversy, is disclaimed; and also that any intention to set a precedent for any mutilation of the records of the Association is disclaimed.

*Resolved*, That we do recognize the meeting of the Georgia Medical Association of 1868, in Augusta, as a regular meeting of that body.

These were adopted. Dr. Battey then offered the following, which was also adopted:

*Resolved*, That the second resolution offered by Dr. Logan, be appended to the official letter of Dr. Boring, of April 10th, 1869, and published by the Secretary in the two medical journals and one newspaper at Atlanta.

The following is the official letter referred to:

"Atlanta, Georgia, April 10th, 1869.

"At a meeting of the Faculty of the Atlanta Medical College, of 1868, held in the city of Atlanta, March 16th, 1869, the following resolution was unanimously adopted, viz:

*Resolved*, That this Faculty disavow any purpose to reflect upon the Medical Association of Georgia, either in the 'Memorial' presented to the Legislature of 1868, or upon any other occasion, and that our Representatives, who may attend the meeting of said Association, to be held in the city of Savannah, on the 14th inst., be and they are hereby instructed to present this disavowal, together with that contained in said Memorial."

By order of the Faculty,

JESSE BORING, M. D.
Dean of the Faculty.

Wm. S. Armstrong, M. D., Secretary.

It is not our desire or purpose to comment on a "settlement" all declare to be satisfactory, and we therefore append extracts from private letters in lieu thereof:

——— ———, April 16th, 1872.

Drs. Powell & Goldsmith.

"*Gentlemen*:— * * * * I am sorry now I did not attend the meeting of the Medical Association in Columbus, * * * but am rejoiced to see that the right has triumphed, as the basis of the settlement (small as it appears), will clearly show to all who are acquainted with the facts, and the record of the four preceding meetings of the Association. And though great wrong may have been committed in the efforts of some (for the last few years), to establish the statements made in the memorial and the resolution passed in Americus, to be true, yet, the action at Columbus, declaring any such intention, is now disclaimed.

I hope you, gentlemen, will accept the situation. You can very well afford to do it. You have lost nothing in the settlement. All the positions taken by you and by those who acted with you, stand approved. If you [illegible]nt right and have not been right, the quarrel is with those who passed the resolution in Columbus. If you do not now occupy an honorable position, then there is no honor in being right, or being so declared by those who composed the Georgia Medical Association, to say nothing of justice, which, in this as in everything else, will do her perfect work." Another correspondent says:

"I see by the papers that the professional troubles, so far as the Association is concerned, were settled at the meeting at Columbus. Our friends have nothing to regret or take back, but I don't know if it would not be best for you, gentlemen, to publish, in THE COMPANION, the memorial and the action of the Association since it met in Augusta in 1868. This would be justice to many who have received wrong impressions, to the injury of their own feelings and the character of some who are now *rectus in curia*, etc."

We thank our friends for their comments. We think, under all the circumstances, we had better withhold, for the present, at least, the publication of the memorial. We do not desire to re-open an old sore—said to be closed (if not healed). We are for peace, when and so long as it can be had and maintained without injury to the cause of truth, and without bequeathing to error a resuscitating element.

